# Facilitating job retention for chronically ill employees: perspectives of line managers and human resource managers

**DOI:** 10.1186/1472-6963-11-104

**Published:** 2011-05-17

**Authors:** Joke A Haafkens, Helen Kopnina, Martha GM Meerman, Frank JH van Dijk

**Affiliations:** 1 Amsterdam Institute for Advanced Labour Studies (AIAS), University of Amsterdam, Plantage Muidergracht 12, 1018 TV, Amsterdam, the Netherlands; 2 Department of General Practice, Academic Medical Center, University of Amsterdam, Meibergdreef 15, 1105 AZ, Amsterdam, the Netherlands; 3 Coronel Institute for Work Environment and Health, Academic Medical Center, University of Amsterdam, Meibergdreef 15, 1105 AZ, Amsterdam, the Netherlands

## Abstract

**Background:**

Chronic diseases are a leading contributor to work disability and job loss in Europe. Recent EU policies aim to improve job retention among chronically ill employees. Disability and occupational health researchers argue that this requires a coordinated and pro-active approach at the workplace by occupational health professionals, line managers (LMs) and human resource managers (HRM). Little is known about the perspectives of LMs an HRM on what is needed to facilitate job retention among chronically ill employees. The aim of this qualitative study was to explore and compare the perspectives of Dutch LMs and HRM on this issue.

**Methods:**

Concept mapping methodology was used to elicit and map statements (ideas) from 10 LMs and 17 HRM about what is needed to ensure continued employment for chronically ill employees. Study participants were recruited through a higher education and an occupational health services organization.

**Results:**

Participants generated 35 statements. Each group (LMs and HRM) sorted these statements into six thematic clusters. LMs and HRM identified four similar clusters: LMs and HRM must be knowledgeable about the impact of chronic disease on the employee; employees must accept responsibility for work retention; work adaptations must be implemented; and clear company policy. Thematic clusters identified only by LMs were: good manager/employee cooperation and knowledge transfer within the company. Unique clusters identified by HRM were: company culture and organizational support.

**Conclusions:**

There were both similarities and differences between the views of LMs and HRM on what may facilitate job retention for chronically ill employees. LMs perceived manager/employee cooperation as the most important mechanism for enabling continued employment for these employees. HRM perceived organizational policy and culture as the most important mechanism. The findings provide information about topics that occupational health researchers and planners should address in developing job retention programs for chronically ill workers.

## Background

Chronic diseases are increasingly prevalent among people of working age in Europe [1,2]. In the Netherlands, about one third of the working population (37%) has a chronic disease [3]. Musculoskeletal, respiratory, gastrointestinal, and cardiovascular conditions are common, as are skin diseases, diabetes, mental health problems, hearing disorders and cancer [4]. Chronic diseases do not go away. Other common characteristics are that they usually require regular medical care and self-management and that they may limit what one can do [5]. Many chronically ill workers (30%-60%) feel more or less restricted by their condition at work [4,6-8], and they are more likely to exit their job prematurely than their healthy colleagues [6,9-15]. Early job discontinuation can lead to negative health and socioeconomic outcomes for the individual [15,16] and increased costs to employers and society [2,10,17]. To reverse these problems, recent EU and national social and employment policies are encouraging employers to facilitate continued employment for employees with long-term health problems and disabilities [16,18,19].

In the Netherlands, the responsibility for the provision of work disability benefits has shifted from the state to the employer in 2002 [3]. In response to this policy shift employers have established new programs to promote job retention for employees who are ill. In the past occupational physicians and occupational health services were mainly responsible for the development and implementation health and safety programs in Dutch companies. Today, line managers (LMs) and human resource managers (HRM) are playing an increasingly important role in workplace health management. A similar development is taking place in other countries [20]. In recent years, many large and medium-sized organizations in the Netherlands have created "social medical teams" at the level of departments, which have the task to implement and monitor organizational health and safety policies and procedures. In addition to an occupational physician, the department's LM and HRM constitute the core members of these teams. At present, in most Dutch organizations job retention programs are mainly focused on reducing the levels and duration of sickness absence. Employees who are on sick leave for more than two weeks are typically required to make a return-to-work plan together with their LM, while occupational physicians and HRM have the role to advice the two parties on feasible job adaptations that may be needed to implement these plans. The available statistics indicate that there has been an over-all decrease in absenteeism and work disability rates in the Netherlands since 2004. At the same time, however, an increase of work disability rates has been observed among employees with some chronic conditions (mental health problems and musculoskeletal disorders) [2,21].

Frank and Cullen have characterized active absence or return-to-work management as secondary prevention, which occurs *after *illness or disability has occurred and aims at reducing long-term disability [22]. Disability research suggests, however, that the retention of chronically ill workers requires a more pro-active approach, combining secondary prevention with primary prevention [23-27]. According to Frank and Cullen, "primary prevention aims at reducing the risk of injury or illness *before *the absence occurs (while the person is still healthy). This aim is generally accomplished by modifying factors known to increase the risk of work disability by directly controlling a specific hazard or set of hazards, for example by increasing a worker's skills or modifying the work environment" [22].

Internationally, occupational health researchers and practitioners have developed models and interventions for workplace based primary and secondary work disability prevention [23,24]. Moreover, in recent years Dutch occupational physicians have developed guidelines for the prevention of work-related problems for a number of chronic conditions, together with general practitioners and medical specialists (e.g., depression, musculoskeletal, respiratory and cardiovascular conditions) [28]. However, several authors have noted that the guidance employees with chronic health conditions is still a largely neglected topic in the professional (human resource) management literature and training [5,29,30].

HRM and LMs have different functions and tasks within the organization. HRM usually occupy a staff function. They have the responsibility to ensure that employees become and remain valuable to the organization. They deal with issues related to people management such as compensation, hiring, performance management, organization development, safety, wellness and disability management, benefits, employee motivation, communication, administration, and training. The role of HRM is changing. In the past they were primarily responsible for personnel management. More recently, HRM departments are also becoming a strategic partner for the chief executives of an organization. In this role, HRM professionals contribute to the development of and implementation of organization-wide business plans, policies and objectives [31].

The LM is primarily responsible for organizing and supervising the work and productivity of a team or a department. Typical tasks of the LM are managing operational costs, providing technical expertise, organizing work allocation and rotas, monitoring work processes, checking quality, dealing with customers/clients, measuring operational performance. At present, also many of the personnel management tasks that were formerly performed by HRM have been devolved to the line manager, such as the recruitment and hiring of personnel, performance appraisal, coaching, resolving disputes and the implementation of health disability management policies [32]. LMs are most likely to receive the first requests for accommodations from employees with health problems. But it has been recognized that the successful implementation of such accommodations requires adequate coordination between LMs and other professionals, including HRM and occupational health services [27,33]. Organizational studies have observed that there may be a gap between what is formally required by an organization's HR policy and what is actually delivered by LMs [34]. For instance, a case study from Cunningham and colleagues in four organizations in the UK found that HRM and LM may have very different perspectives and practices as regards to the protection of job-security for workers who are on sick leave. More concretely, the authors observed that LMs were not always able to comply with HR guidelines in this area, because they were confronted with conflicting policy requirements, weaknesses in training, lack of support from relevant human resources and occupational health specialists and various work and budgetary pressures [33].

Occupational health researchers and practitioners have argued that the retention of chronically ill employees requires a pro-active approach, combing primary prevention, with active absence management (secondary prevention). LMs and HRM are increasingly important stakeholders in workplace health and disability management. Disability researchers have shown that each stakeholder group may have its own perspective on disability management issues and that the perspectives of all stakeholders need to be taken into account if programs are to be effective [34,35]. Thus far, little is known about the perspectives of HRM and LMs on what is needed to facilitate continued employment for chronically ill employees. Given their different roles in the organization, these perspectives may differ [33,36]. Towards this background, the aim of this study was to explore and compare the perspectives of Dutch LMs and HRM on what is needed at the workplace to facilitate job retention for chronically ill employees.

## Methods

Because there has been no previous research on the perspectives of HRM and LMs on factors that may facilitate job retention for chronically ill employees, the use of qualitative methods to elucidate their viewpoints was warranted [37]. In this study we selected concept mapping as a research method because it is a structured qualitative approach that can be used in small groups to elicit and map out ideas from participants about complex issues [38,39]. The concept mapping methodology typically consists of the following steps: In the *preparation step*, the research team decides on a focus question. In the *generation step*, participants are asked to develop a set of statements (ideas) that address the focus. In the *structuring step*, participants are asked to structure their ideas by sorting the statements into categories that make sense to them and by rating them according to importance. During the *representation step *the first analysis is done. This is the process of taking the sort and rating input and "representing" it in map form. In the fifth step, *the interpretation step*, the researchers stimulate the participants to develop their own labels and interpretations for the various maps. In this study, Concept Systems software version 4.0 was used to support data entry and analysis [40].

### Participants

Qualitative research is aimed at acquiring in-depth knowledge of certain phenomena and not at statistical generalization. In order to achieve this, qualitative researchers typically recruit small samples of "information rich" cases, in accordance with the purpose of their study [41,42]. In general, samples 10-20 participants are regarded as adequate for achieving data saturation in qualitative research [43]. In previous concept mapping studies, small groups of 10-20 participants have proven to be sufficient for developing concept maps [38,39,44,45]. Based on these considerations, our aim was to recruit a minimum of 10 HRM and 10 LMs. We sought candidates with at least 2 years of professional experience and an "ordinary" professional profile. Therefore, HRM and LMs who were known experts in disability management were not eligible. Participants were recruited through a large institution for higher professional education (2000 employees) and an occupational health services (OHS) organization in the Netherlands. In each organization, the research committee invited a contact person to locate suitable candidates. In the first organization this contact person was a member of the HRM department, and in the second an occupational physician. In each organization we wanted to locate an equal number of HRM and LMs. At the institution for higher education candidates were sought among the personnel of the organization. At the OHS organization candidates were sought among the personnel of companies that were served by this organization. In each organization 20 potentially suitable candidates were invited via e-mail. Fifteen individuals agreed to participate at the institution for higher education (9 HRM and 6 LMs), and 12 agreed at the OHS organization (8 HRM and 4 LMs). The latter worked for a variety of companies in the transportation, ICT, financial, industrial and services sector. The main reason given for non-participation was time restrictions.

### Data-collection

Concept mapping starts with a focal question. In consultation with two HRM and two OHS professionals, the following fill-in-the-blank focal question was formulated for this study: *In order to ensure that chronically ill employees can continue to work, it is necessary that ...? *At each recruitment site, one concept mapping meeting was held, led by a facilitator and an assistant. At the institution for higher education, the meeting started with a 50-minute brainstorming session during which participants were asked to formulate statements to complete the focal question. The concept mapping method requires that each statement is clear and reflects only one distinct idea. Therefore, the facilitator encouraged participants to clarify jargon, helped to edit the statements, and eliminated statements expressing similar ideas. The brainstorm session ended when saturation was reached [45], and no new ideas could be elicited from the participants. All statements were typed out on a computer and printed on separate cards. After a break, the participants received a complete set of cards. They were first asked to rate the statements on their importance to strategies for ensuring that chronically ill employees can continue to work, using a five-point Likert scale, with 1 indicating unimportant and 5 indicating extremely important. They were then asked to sort the statements logically into groups according to themes and to provide a name for each group (cluster) of statements. Participants performed these rating and sorting tasks individually. Subsequently the individual scoring forms were entered into a computer and preliminary results generated by the concept mapping software [40]. The computer software suggested various names or labels for the clusters, based on the names the individual participants had assigned to groups of statements. In a final group discussion, participants were given the opportunity to review and discuss the cluster maps and priority ratings and to suggest a name for each cluster which they regarded most appropriate as a group. Clusters for HRM and LMs and for the whole group were presented and discussed separately.

The statements generated at the first meeting were also the basis for the concept mapping at the OHS organization. Here, participants were only invited to rate and sort these statements and to discuss the preliminary results (concept maps) according to the procedure described above. Meetings were held in April and June 2007 and lasted 5 and 4 hours, respectively. Two respondents who were not able to attend the meetings provided the results of the sorting and rating tasks by mail.

### Data-analysis

The results from the two concept mapping meetings were analyzed separately for HRM and LMs, using Trochim's methods [38,39]. First, the means of the importance ratings the participants assigned to each statement were calculated at a group level. This resulted in a rated list of statements for HRM and LMs. Secondly, using a series of statistical analyses (multi-dimensional scaling techniques [46] and then hierarchical cluster analysis [38,39]), the participants' statements and sorting results were aggregated at a group level. The result was a conceptual map for LMs and HRM. On these maps statements that were more often placed under the same theme by the group members are located closer to each other. As described above, in each concept mapping session the maps were discussed with the participants. The researchers selected the final number of clusters, based on the results from both sessions. Average importance ratings were computed for each cluster and labels were assigned to them based on the names proposed by the participants. To identify similarities and differences between the perspectives of HRM and LMs, the researchers compared the content and the importance ratings of the thematic clusters that were identified by the two groups.

### Ethics

This study used a social scientific method to explore the perspectives on workplace health management among HRM and managers who volunteered to participate in the study. According to the Dutch Medical Research Involving Human Subjects Act (WMO) a study must undergo a medical ethics review if it is medical/scientific research and people are subjected to procedures or are required to follow rules of behavior. Social scientific research does not fall within the scope of this law, unless either the frequency with which subjects are asked to complete a questionnaire are sufficient to bring about a temporary change in the subject's lifestyle or the nature of the questions is such that the subject could be regarded as having received a particular treatment or having been asked to behave in a particular way. (See, http://www.ccmo-online.nl/main.asp?pid=10&sid=30&ssid=51) For that reason, the present study was exempt from review by the medical ethics committee of the Academic Medical Centre of the (AMC) of the University of Amsterdam or any other authorized medical ethics committee in the Netherlands. However, by way of good research practice, the researchers have followed the recommendations with regard to respect for human subjects involved in medical research from the "*AMC research code independence in scientific research*" [47]. Concretely, potential participants were informed about the purpose, the procedures and implications of participating in the concept mapping sessions through an invitation letter. From those who decided to participate, written informed consent was acquired prior to participation, both for joining the concept mapping sessions and the publication of the results, provided that data protection rules would be applied when storing, analyzing and reporting personal data or information that is deducible to the participants. Participants were given the opportunity to withdraw their contribution at any time and they have been informed about the results of the study.

## Results

Twenty-seven professionals participated in the study: 17 HRM (63%) and 10 LMs (Table 1). One-third of the participants reported they had received some kind of training in the management of employees with ill health. Of the 13 participants who had worked with employees with a chronic illness, 11 reported they had encountered difficulties in doing so.

**Table 1:** Characteristics of the participants

Characteristic	N	%
		

Gender		

Male	12	(44%)

Female	15	(56%)

Function		

HRM	17	(63%)

Line manager	10	(37%)

Years of experience in function		

Average	8.76 years, SD 7.43	

Work sector		

Higher education	15	(56%)

Other	12	(44%)

Received training in employee health management		

Yes	8	(30%)

No	19	(70%)

Has practical experience in managing chronically ill employees		

Yes	13	(48%)

No	14	(52%)

Has experienced obstacles in managing chronically ill employees		

Yes	11	(85%)

No	2	(15%)

Not applicable	14	

The brainstorm session yielded 35 statements expressing the thoughts of the participants on what is necessary in order to ensure that chronically ill employees can continue to work. The four statements with the highest mean ratings (1 = low, 5 = high) for LMs were: "Mutual trust between employees and managers" (4.4); "The organization should reflect on what it means to be a good employer for chronically ill employees" (4.2); "Chronically ill employees must make decisions for themselves" (4.1); "An employer must be able to make demands on chronically ill employees" (4.1). The four statements with the highest mean ratings for HRM were: "Mutual trust between employees and managers" (4.5); "Good contact between manager and employee" (4.4); "The employer must realize that the employee should not continue to work in a situation that is no longer healthy" (4.4); "There must be good contact between manager, occupational physician and employee" (4.3).

Analysis of the sorting activity revealed that each group sorted the statements into a set of six distinct thematic clusters, referring to conditions they perceived as necessary to ensure that chronically ill employees can continue to work. Tables 2, 3 describe the clusters and the corresponding statements with median priority ratings for the clusters and mean priority ratings for the items, for LMs and HRM respectively. (Additional File 1 Figure S1 and Figure S2 depict the concept maps for LMs and HRM, respectively.)

**Table 2:** Perspectives of line managers on what is needed to ensure that chronically ill employees can continue to work: clusters, statements and mean priority ratings. *

Clusters/statements	Cluster median/Item mean scores
Cluster 1. Good cooperation between manager and employee	3.9
	
1. Mutual trust between the manager and the employee.	4.4
2. An employer must be able to make demands on chronically ill employees.	4.1
3. Good contact between manager and employee.	3.9
4. A manager who creates time and space to listen to chronically ill employees.	3.8
5. A manager and an employee who share responsibility for (the employee's) employability.	3.8
	
Cluster 2. Managers must have knowledge about impact of disease on work	3.6
	
1. The manager must know the difference between chronic illness and sick leave.	3.9
2. The manager must know the work-related risk factors for the employee.	3.7
3. Employees' fear of negative consequences must be alleviated.	3.7
4. The employer must realize that the employee should not continue to work in a situation that is no longer healthy.	3.7
5. A manager must know which options s/he has to facilitate optimal job performance.	3.7
6. A manager must have knowledge about the disease to be able to act proactively.	3.5
7. A manager must be aware of the meaning of the medical diagnosis.	3.2
	
Cluster 3. Employees must accept responsibility	3.6
	
1. Chronically ill employees must make decisions for themselves.	4.1
2. Managers must also be concerned with the consequences of the (employee's) illness for his or her colleagues.	3.9
3. Chronically ill employees must not set one-sided limits.	3.8
4. Chronically ill employees must not be ashamed to talk about their condition.	3.8
5. Chronically ill employees must understand the capabilities and limitations of their colleagues.	3.6
6. Chronically ill employees must not conceal their illness.	3.5
7. Chronically ill employees must be open about their condition with colleagues.	3.3
8. Chronically ill employees must realize that privacy is not always possible.	3.3
	
Cluster 4. Work accommodations	3.6
	
1. The work/job must be matched to the employee's condition.	3.9
2. Suitable work must be sought.	3.6
3. As much as possible, the needs of the employee should be met, taking into account the organization's capabilities.	3.6
4. Chronically ill employees need to be given guidance.	3.3
	
Cluster 5. Information and knowledge transfer within the company	3.2
	
1. There must be good contact between manager, occupational physician and employee.	3.5
2. The personnel officer must know the difference between chronic illness and sick leave.	3.4
3. Personnel officers must know the employees who have a chronic illness.	3.0
4. The company health service must know the employees who are ill.	2.7
	
Cluster 6. Company policy	3.1
	
1. The organization should reflect on what it means to be a good employer for chronically ill employees.	4.2
2. There must be more openness about this topic within the organization.	3.3
3. The organization needs to create an in-house resource with specific information about chronic illness.	3.1
4. The organization needs to pay attention to best practices in this area.	3.0
5. The HRM department needs to implement adaptations for chronically ill workers (elevator, wheelchairs).	2.9
6. The organization needs to come to a clear agreement about its norms regarding chronically ill employees.	2.9
7. The organization needs to create a focal point with specific expertise regarding chronically ill employees.	2.6

*Lengthy statements have been rephrased.

**Table 3:** Perspectives of human resource managers on what is needed to ensure that chronically ill employees can continue to work: clusters, statements and priority ratings.*

Clusters/statements	Cluster median/Item mean scores
Cluster 1. Company policy	4.1
	
1. The employer must realize that the employee should not continue to work in a situation that is no longer healthy.	4.4
2. The work/job must be matched to the employee's condition.	4.3
3. The employer must be able to make demands on chronically ill employees.	4.1
4. Managers must also be concerned with the consequences of the employee's illness for his or her colleagues.	4.1
5. The organization should reflect on what it means to be a good employer for chronically ill employees.	4.1
6. The organization needs to come to a clear agreement about its norms regarding chronically ill employees.	3.9
7. As much as possible, the needs of the employee should be met, taking into account the organization's capabilities.	3.7
8. Chronically ill employees must understand the capabilities and limitations of their colleagues.	3.6
	
Cluster 2. Culture of trust and openness	3.9
	
1. Mutual trust between manager and the employee.	4.5
2. Good contact between manager and employee.	4.4
3. There must be good contact between manager, occupational physician and employee.	4.3
4. A manager who creates time and space to listen to chronically ill employees.	3.9
5. Chronically ill employees must not conceal that they are ill.	3.6
6. There must be openness about this topic within the organization.	3.5
7. Chronically ill employees must be open with colleagues about their condition.	3.4
	
Cluster 3. Shared responsibility	3.8
	
1. The manager and the employee need to share responsibility for the employee's employability. 2. The employee's fear of negative consequences should be alleviated.	4.2 3.9
3. Chronically ill employees must make their own decisions.	3.8
4. Chronically ill employees must not be ashamed to talk about their condition.	3.8
5. Chronically ill employees must not set one-sided limits.	3.7
6. Chronically ill employees must realize that privacy is not always possible.	3.3
	
Cluster 4. Managers and personnel officers must have knowledge of chronic disease and its impact on work.	3.6
	
1. The manager must know the work-related risk factors for the employee.	4.1
2. The manager must know what options s/he has to facilitate good job performance.	4.0
3. The manager must know the difference between chronic illness and sick leave.	3.6
4. The personnel officer must know the difference between chronic illness and sick leave.	3.5
5. The manager must have knowledge about the disease to be able to act proactively.	3.5
6. The manager must be aware of the meaning of the medical diagnosis.	3.4
7. The personnel officers must know the employees who have a chronic illness.	3.1
	
Cluster 5. Work adaptations	3.5
	
1. Suitable work is being sought.	3.8
2. The company health service must know the employees who are ill.	3.6
3. The HRM department must implement adaptations for chronically ill workers (elevator, wheelchairs).	3.5
4. The organization needs to pay attention to best practices in this area.	3.3
	
Theme 6. Support services within the company	2.9
	
1. Chronically ill employees need to be given guidance.	3.4
2. The organization needs to create a focal point with specific expertise regarding chronically ill employees.	2.7
3. The organization needs to create an in-house resource with specific information about chronic illness.	2.5

* Lengthy statements have been rephrased

### Line Managers (Table 2)

LMs regarded "good cooperation between manager and employee" as the most important condition to ensure continued employability for chronically ill employees (Cluster 1). The statements grouped under this theme indicate that this involves mutual trust, contact, shared responsibilities between manager and employee, and attentiveness from the manager, but also the ability of the employer to make demands on the employee. Cluster 2 indicates that LMs also find it important that "a manager must have basic knowledge of how chronic illness can affect work". A relatively high priority was also assigned to the "role of employees themselves" (Cluster 3). For instance, employees should make their own decisions, be aware of the limitations and potential of their colleagues and be open about their condition. Almost just as important is the theme that "work should be accommodated to the condition and needs of the employee", within the capabilities of the organization (Cluster 4). Cluster 5 indicates that "good information and knowledge transfer between managers, occupational physicians and HRM "is also perceived as a prerequisite for facilitating the employability of chronically ill workers. Cluster 6 concerns the need to develop a "company policy" with respect to chronically ill employees. Although this theme has the lowest average score (3.1), a relatively high score was assigned to the statement "an organization should reflect on what it means to be a good employer for the chronically ill employee." Other statements grouped under this theme refer mostly to organizational policies and practices that need to be in place according to the LMs.

### HRM (Table 3)

HRM assigned the highest importance to "company policy" as a condition to facilitate sustained employability for chronically ill employees (Cluster 1). The statements they grouped under this theme refer to what the employer, the manager, the organization and the employees must do in order to develop such a policy: the *employer *must realize that the employee cannot continue to work in an unhealthy situation; the work must be suited to the condition of the employee; *managers *must have the right to make demands on employees and evaluate the consequences of an employee's illness for his or her colleagues; the *organization *needs to reflect on what good employership involves; and the e*mployees *must understand their capabilities and limitations. A second, almost equally important cluster is that there must be "a culture of trust, openness and communication" within the organization. This theme contains statements regarding the relationship between managers and employees. The third cluster indicates that HRM also feel that chronically ill employees and managers must "share responsibilities" in order to ensure continued employability for the employee. From the part of the LMs this means removing the employee's fear of repercussions or shame about his or her condition, while the employee must realize that privacy is not always possible. The fourth cluster contains statements indicating that both "managers and personnel officers" should have sufficient "knowledge about chronic illness and its impact on work" to be able to act proactively. The fifth cluster indicates that "work adaptations" are also seen a condition to help ensure job retention for chronically ill employees, and the statements in this cluster refer to who should be responsible for providing these accommodations. Cluster 6, "support services within the company", was given the lowest average priority. It contains some low-rated statements suggesting possibilities for centralized services for chronically ill employees within the organization.

### Comparison

Comparison of the clusters shows that both groups identified four rather similar thematic clusters referring to conditions that are necessary in order to ensure that chronically ill employees can continue to work: managers must be knowledgeable about the impact of chronic disease on the work situation (Cluster 2 LMs, Cluster 4 HRM); employees must accept responsibility (Cluster 3 LMs, Cluster 3 HRM); work adaptations (Cluster 4 LMs, Cluster 5 HRM) and company policy (Cluster 6 LMs, Cluster 1 HRM). But if we look at the content of these clusters, there are some interesting differences. For instance with respect to knowledge, LMs only include sentences referring to themselves, while HRM also refer to the importance of knowledgeable HRM. In the thematic cluster referring to "responsibilities of employees", HRM focus on "shared" responsibilities between managers and employees, but LMs refer only to the employees. Moreover, the sentences grouped under the cluster company policy show that both group seem to have diverging views of the issues that relate to company policy.

Two themes were identified only by LMs:" good cooperation between the managers and employees" (Cluster 1); and "information and knowledge transfer within the company" (Cluster 5). Two other themes were identified solely by HRM professionals: "a culture of trust, openness and communication within the organization" (Cluster 2) and the low-rated cluster "support within the organization" (Cluster 6).

A comparison of the priority ratings of the clusters suggests that LMs and HRM differ in their perspectives (conceptual frameworks) with regard to the mechanisms through which job retention strategies can be implemented. For LMs, good manager/employee collaboration (Cluster 1) seems to be the most important entry point for accommodating work to the capabilities of the employees and the expectations of the organization (Cluster 4). This requires both managers who have sufficient knowledge regarding the management of employees with a chronic disease (Cluster 2) and employees who assume responsibility for decisions regarding their work, including the disclosure of their condition (Cluster 3). Information and knowledge transfer within the company (Cluster 5) and company policy (Cluster 6) are seen as additional, less important steps. In contrast, for HRM, company policy (Cluster 1) and company culture (Cluster 3) seem to be the most important entry points to enable job retention for chronically ill employees. A company culture that allows for openness and trust could facilitate shared responsibility on the part of employees and managers (Cluster 3) and enable managers and personnel officers to gain sufficient knowledge regarding the management of chronically ill workers (Cluster 4). These steps can then lead to the design or other types of support within the organization (Cluster 6) or directly to work accommodations (Cluster 5).

## Discussion

In this qualitative study concept mapping proved to be a useful method to elicit perspectives of HRM and LMs on what is needed to facilitate continued employment for chronically ill workers. The brainstorming session allowed participants to generate their ideas about this issue. The rating and sorting procedures gave them an opportunity to structure those ideas.

We found both similarities and differences between the views of LMs and HRM on what may facilitate job retention for chronically ill employees. Mutual trust between the manager and the employee was rated as the most important statement by both groups. Four thematic clusters were mentioned by both groups; two uniquely by LMs; and two uniquely by HRM. LMs saw "good employee/manager cooperation" as the most important starting point for enabling job retention. For HRM the most important starting point was "corporate policy and culture". This may not be surprising, given the differences in professional responsibilities and job content between the two groups.

The strength of our study is that it builds directly on the experiences and ideas of immediate stakeholders. The study has also limitations. One limitation is that concept mapping allows the inclusion of only a limited number of participants [38]. In this study our selection was limited to 10 LMs and 17 HRM who worked in a variety of medium- or large-sized Dutch companies. About one third of the participants had received some kind of training in managing employee health. However, the career effects of chronic illness had not been addressed during this training. Indeed, most respondents indicated during the meeting that they did not know how many people at their own workplace were living with a chronic health problem. This corresponds with findings from a qualitative study on sickness absence management among LMs and HRM in the UK by Munir et al. [48]. Most participants said during first the group session that they joined the project because they wanted to learn more about chronic disease management at the workplace. It is possible that HRM and LMs with more experience in disability management would have generated other statements and concept maps. It should also be noted that the list of statements used for the rating and clustering procedures was generated by participants of the first concept mapping meeting, all of whom worked for an institution of higher education. It is possible HRM and LMs working in other contexts would have created other statements because culture differences between companies can be important. These specific characteristics of the study population should be kept in mind in interpreting the results. Furthermore, we saw that the mean priority ratings of the thematic clusters were very close to each other, varying from 3.9 to 3.1 for LMs and from 4.2 to 2.9 for HRM. In interpreting these results it should be kept in mind that the concept mapping methodology uses the rating of statements and clusters mainly as a way to support participants in structuring their ideas, and not for making statistical inferences. We also observed that all the statements were regarded as rather important (mean ratings were ≥ 2.5 on a five point scale). This may be understandable, because the statements were constructed by participants themselves. However, the method of data collection and analysis were rigorous and consistent, and the results are plausible in the light of the available literature on chronic disease management.

This study is unique in that it focuses on perspectives of LMs and HRM on what is needed to facilitate job retention for chronically ill employees. Most studies in this area have focussed on the perspectives and practices of LMs and HRM in disease absence or return-to-work management (secondary work disability prevention) [27,33,35,48,49]. James et al. [50] proposed an evidence-based conceptual framework for organizational dynamics that may enhance the return-to-work and job retention of ill workers. Several of the concepts that are included in this framework were also identified by our study participants (e.g., the establishment and implementation of a policy framework, the provision of rehabilitative support and coordination of the rehabilitative process). Other concepts from James' framework were not identified by the study participants (e.g., speedy identification of vulnerable workers, rights of workers to be represented by worker representatives (e.g., labour union) and the monitoring and review of existing policies). Many studies have identified the LM as the most central link between the organization and the employee and, consequently, as the most central stakeholder in workplace disability management [23,24,35,48,49]. A study by Cunningham et al. [33] indicated that LMs tend to take the responsibility for disability management on their own shoulders, without asking support from occupational or HRM services. In the present study a somewhat similar approach was reflected in the concept map that was generated by the LMs. They perceived "good cooperation between manager and employee" as the most important condition enabling job retention for chronically ill employees. The study by Cunningham et al. [33] emphasized the need for employers to provide adequate training to LM's in worker's health and disability management. This theme was also reflected in the concept maps of the LMs and HRM in this study. Corroborating with the views of HRM in the present study, studies on occupational disease and injury prevention and management have called attention to the importance of organizational level determinants (e.g., company culture and managerial style) as a facilitating factor for performing these tasks [23,24,51,52]. Finally, some of the conditions which LMs and HRM perceived to be necessary for ensuring job retention for chronically ill workers are consistent with those identified by other stakeholders. For instance, previous studies found that chronically ill employees themselves and physicians also emphasized the importance of the presence of managers who are knowledgeable about the impact of chronic disease on the work situation, employees who take responsibility for keeping their job, good cooperation between the managers and employees, and collaboration with the occupational physician, and timely work adaptations, be it in a somewhat different terminology [43,44,53].

LMs and HRM are playing an increasingly important role in workplace health management. This concept mapping study provided insight into the perspectives of Dutch LMs and HRM on what is needed to facilitate continued employment for chronically ill employees. The extent to which these perspectives are shared by LMs and HRM working in other countries or organizational settings remains to be determined through other studies. Despite this caveat, we believe that the study provides information about topics that occupational health researchers and planners should take into consideration when developing job retention programs for chronically ill workers. Occupational health research has shown that work accommodations which have proven to be effective in facilitating the employability of chronically ill employees are not always applied at the workplace [e.g., [7]]. The statements and thematic clusters that have been identified in this study may be useful to explore factors that may inhibit or facilitate the application of such work accommodations by LMs and HRM. The study also offers some specific suggestions for occupational health practitioners. Within organizations, they have the task of to develop, supervise, implement or evaluate company health programs, but they also consult LMs and HRM on the guidance of individual employees. This study indicates that LMs and HRM view knowledge about the impact of chronic disease on the work situation as one of the basic requirements for guiding chronically ill employees. It could therefore be a task for occupational physicians to provide this information to these professionals, e.g. through in-company training.

## Conclusions

This study identified both similarities and differences between the perspectives of Dutch LMs and HRM on conditions that may facilitate job retention for chronically ill employees. LMs and HRM identified four similar conditions: LMs and HRM must be knowledgeable about the impact of chronic disease on the employee; employees must accept responsibility for work retention; work adaptations must be implemented; and clear company policy. Two conditions were only identified by LMs: good manager/employee cooperation and knowledge transfer within the company. Unique conditions identified by HRM were: a company culture that facilitates trust and openness and organizational support. LMs perceived manager/employee cooperation as the most important mechanism for enabling continued employment for these employees. HRM perceived organizational policy and culture as the most important mechanism. These findings provide information about topics that occupational health researchers, planners and practitioners should take into consideration when developing job retention programs for chronically ill workers.

## Abbreviations

LM: Line Manager; HRM: Human Resource Manager; HR: Human Resources; OHS: Occupational Health Services.

## Competing interests

The authors declare that they have no competing interests.

## Authors' contributions

JH designed and conducted the study, analyzed the data and wrote the paper. HK critically reviewed several drafts of the paper. MM helped to design and conduct the study, the interpretation of the data and she critically reviewed several drafts of the paper. FvD contributed the data analysis and interpretation and the drafting of the paper. All authors read and approved the final manuscript.

## Pre-publication history

The pre-publication history for this paper can be accessed here:

http://www.biomedcentral.com/1472-6963/11/104/prepub

## Supplementary Material

Additional file 1 **Concept maps**. Figure s1 shows the cluster map indicating perspectives of line managers on what is needed to ensure continued employment for chronically ill employees. Figure s2 shows the cluster map indicating perspectives of HRM on what is needed to ensure continued employment for chronically ill employees. Click here for file
