# Supplementary material for: Facilitating job retention for chronically ill employees: perspectives of line managers and human resource managers
Source: BMC Health Serv Res. 2011 May 17;11:104. doi: 10.1186/1472-6963-11-104 (PMC3118107; doi:10.1186/1472-6963-11-104)

Concept maps for line managers and HRM

Figure 1- Perspectives of line managers on what is needed to ensure continued employment for chronically ill employees: cluster map.


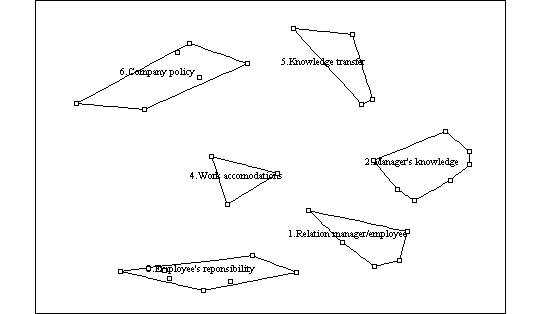


Figure 2- Perspectives of human resource managers on what is needed to ensure continued employment for chronically ill employees: cluster map


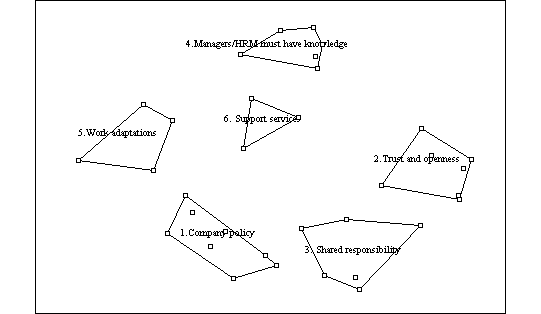

Supplement: Additional file 1 — Concept maps. Figure s1 shows the cluster map indicating perspectives of line managers on what is needed to ensure continued employment for chronically ill employees. Figure s2 shows the cluster map indicating perspectives of HRM on what is needed to ensure continued employment for chronically ill employees. [file 1472-6963-11-104-S1.DOC]
